# Benefits from sketching for improving comprehension monitoring from illustrated texts

**DOI:** 10.3758/s13423-025-02730-5

**Published:** 2025-09-02

**Authors:** Jennifer Wiley, Tim George, Thomas D. Griffin

**Affiliations:** Department of Psychology, University of Illinois at Chicago, 1007 W. Harrison St (MC285), Chicago, IL 60607 USA

**Keywords:** Comprehension, Diagrams, Expository text, Metacomprehension, Multimedia

## Abstract

Although frequently used with instructional expository text, it has been suggested that illustrations can lead to illusions of understanding (beliefs that we understand better than we actually do). In this study using geoscience texts, relative metacomprehension accuracy (the ability to monitor one’s own understanding across a set of topics) was found to be particularly poor when only some topics were illustrated. However, when readers were prompted to generate sketches while reading, relative accuracy was improved, and was more similar across illustration conditions. Consistent with the situation-model approach to metacognition, sketching activities may help readers to generate valid and diagnostic cues on which to base their judgments of understanding and avoid reliance on heuristic cues or superficial processing.

Although textbook illustrations are meant to improve comprehension of concepts from expository science texts, their presence may alter perceptions of understanding (Cardwell et al., 2017), and lead to poor accuracy in gauging understanding across topics (Ikeda et al., 2013; Jaeger & Fiorella, 2024; Jaeger & Wiley, 2014; Serra & Dunlosky, 2010; Wiley et al., 2017). Readers are generally poor at gauging how well they have understood what they have read in expository science texts, and many people experience illusions of understanding for explanations of scientific phenomena (i.e., they believe that they understand phenomena better than they actually do; Rozenblit & Keil, 2002). Readers also typically exhibit poor relative metacomprehension accuracy, which means they are poor at judging which topics they have understood the best or worst among a set of topics (Dunlosky & Lipko, 2007; Griffin et al., 2019a; Maki, 1998b; Thiede et al., 2009). The present study tests whether relative accuracy may be especially poor when readers are presented with textbook excerpts that include illustrations for only some topics, and whether sketching activities might improve relative accuracy.

The main focus of this study is exploring causes of inaccurate comprehension monitoring as assessed by measures of relative accuracy. The prevailing theory that has been proposed to explain inaccurate comprehension monitoring is the situation-model approach to metacomprehension (Griffin et al., 2019b; Wiley et al., 2016b). This framework is based on two distinct literatures. It pairs metacognitive theories of cue utilization as a basis for making accurate *memory* monitoring judgments (Koriat, 1997) with theories of *text comprehension* (Kintsch, 1994). According to Koriat’s (1997) cue-utilization framework, accurate monitoring is an inferential process that relies on the use of valid cues. According to theories of text comprehension, to provide accurate predictions for future performance on comprehension tests, these cues need to be based in the situation model (the reader’s representation of what the text is about, including inferences that are not presented in the text) rather than more superficial memory representations for exactly what was read (Rawson et al., 2000; Thiede et al., 2003, 2010; Wiley et al., 2005).

Recent reviews have documented support for the situation-model approach to metacomprehension (Griffin et al., 2019a; Prinz et al., 2020; Wiley et al., 2016b; Yang et al., 2023) as generation activities (such as self-explanation) that prompt readers to access their model of what the text is about generally lead to more accurate metacognitive judgments than more passive activities such as re-reading (Griffin et al., 2008, 2019b). Similar to the present study, most work on this topic has explored relative accuracy in college samples, although there is some work in younger samples as well.

## Effects of illustrations on metacomprehension accuracy

It is common for instructional texts in science courses such as biology, chemistry, and geoscience to include images alongside verbal descriptions of processes, systems, or phenomena. This practice is consistent with theoretical assumptions and prior work showing that spatial thinking and visualizations (non-text-based representations including pictures, drawings, diagrams, and animations) can be supportive of better understanding in science (Ainsworth & Loizou, 2003; Butcher, 2006; Mayer, 1989; Uttal & Cohen, 2012). Although incorporating illustrations into expository science texts is intended to support better instructional outcomes, this benefit is not always realized, as images can cause seduction effects that undermine learning (Butcher, 2006; Harp & Mayer, 1998; Sanchez & Wiley, 2006). This prompts the question of whether the presence of even relevant illustrations (that depict information germane to understanding a process, system, or phenomenon) might affect monitoring and lead to poor discrimination of what is less versus more understood.

There are several possible ways that the presence of images might undermine metacomprehension accuracy. The first is that readers may rely on heuristics, such as a multimedia heuristic (Jaeger & Wiley, 2014; Serra & Dunlosky, 2010) and assume that their understanding will be better whenever an illustration is presented with a text. A second possible reason is that a diagram depicting a complex process might prompt a sense of fluency or a false sense of understanding, especially if the student only processes the image in a rather superficial manner (Butcher, 2006; Jaeger & Fiorella, 2024; Renkl & Scheiter, 2017; Wiley, 2019). Both of these hypotheses could be used to predict that JOUs might be higher, or more overconfidence may result, from the presence of illustrations. However, the results from studies that have tested whether the presence of illustrations has an impact on judgments of knowing, beliefs about learning, and perceptions of understanding fail to offer strong support for these hypotheses (Cardwell et al., 2017; Ikeda et al., 2013; Jaeger & Wiley, 2014; Serra & Dunlosky, 2010; Wiley, 2019; Wiley et al., 2017). Cardwell et al. (2017) found that photos of objects increase judgments of knowledge about how objects work, but these studies did not include texts (participants only saw photos along with the object names, or just the object names). Another study using a short text on lightning (Serra & Dunlosky, 2010) found increased JOU magnitudes when the text was accompanied by six conceptually relevant illustrations, but not increased overconfidence, because the illustrations also improved test performance. Ikeda et al. (2013) employed a similar methodology and found increased JOUs when six brain images were included in a short text about depression. However, a second experiment comparing those brain images to bar graphs containing the same conceptual information found that only the brain images increased JOUs. Similarly, Wiley et al. (2017) found that different types of images led to different patterns of JOUs. Among studies using longer texts and manipulating the presence of a single conceptual illustration, several studies have found no effect on judgments or overconfidence (Jaeger & Fiorella, 2024; Jaeger & Wiley, 2014; Wiley, 2019). These results suggest that students do not simply apply a general heuristic about illustrations that inflates average judgment magnitude and therefore confidence bias whenever illustrations are present. Even if most people endorse a general statement about the utility of textbook images (Serra & Dunlosky, 2010), this does not seem to consistently manifest in their actual judgments related to particular images and their accompanying texts.

In contrast, there does not need to be a uniform impact from the presence of illustrations on JOUs for there to be a negative impact on relative accuracy, which is a measure of how well a reader can discriminate between the topics they understand well from those that they understand poorly. Relative accuracy has become a key measure of metacomprehension precisely because it does not rely upon the average magnitude of either judgments or test performance, and relative accuracy results often diverge from measures of overconfidence based upon the same judgments (for a review, see Griffin et al., 2019a). When images are presented alongside actual texts, the illustrations may introduce invalid cues and idiosyncrasies of particular illustration-text combinations may come into play. Different images may lead to different perceptions of fluency or utility for understanding, and that may lead to inconsistent effects on JOUs (in contrast to a simple overall inflation of JOUs across topics). Further, it is not yet clear whether illustrations offer features that might be leveraged to help readers to better gauge their understanding of each topic, resulting in better relative accuracy, or whether they introduce features that may mislead readers, resulting in poorer relative accuracy. The results from four prior attempts testing for an effect of illustrations on relative accuracy have been mixed. One study failed to observe an effect of illustrations on relative accuracy (Jaeger & Wiley, 2014), while three other studies tended to observe a negative effect, but the effects were small and only significant in one of those studies (Jaeger & Fiorella, 2024).

A common feature of all four of those studies is that the illustration conditions were always all or none (between subjects), without a condition in which a single reader was shown illustrations for only some of the texts. Not only is the inconsistent presence of illustrations across topics more realistic of what students will encounter as they study for classes (not every sub-topic in their textbooks will have illustrations), but it is also the situation that could be most likely to impact relative accuracy. Relative accuracy requires tracking variations in understanding from text to text. It is less sensitive to general heuristics and more sensitive to variations in text-specific features that generate metacognitive cues (Griffin et al., 2019a). The presence of images for only some topics could make it more difficult for readers to track their understanding of each topic. When only some texts in a set are illustrated, this may further undermine accurate metacomprehension if the reader relies on invalid cues including heuristics about the effectiveness of multimedia, and perceptions of fluency or utility, or when the reader engages in superficial processing to guide their judgments of understanding (JOUs) instead of engaging in attempts to test their own understanding of a process or system. Thus, when a set of readings includes both illustrated and non-illustrated texts, the effects from invalid cues may be exaggerated and this could be expected to lead to poor relative accuracy.

## Effects of drawing concept maps or sketches on metacomprehension accuracy

In instructional contexts, students are often asked to draw concept maps or sketches as they learn (Fiorella & Zhang, 2018; Van Meter & Garner, 2005). A general advantage of drawing or sketching is that it prompts constructive processing and active integration of information (Bobek & Tversky, 2016; Hall et al., 1997; Jaeger et al., 2018; Scheiter et al., 2017; Schwamborn et al., 2010; Van Meter & Garner, 2005). These constructive learning activities, like explanation tasks, encourage readers to generate inferences or causal connections between ideas presented across separate sentences, and use their prior knowledge to construct coherent and accurate situation models. Presenting illustrations to readers may prompt information to be processed more passively or may lead to a reduced sense of difficulty, a stronger sense of fluency, a greater ease of processing, or similar subjective reactions. These cues may not be diagnostic of their actual understanding. In contrast, activities that require students to generate diagrams or concept maps may allow for access to cues that better reflect understanding of the topic, and allow students to notice incoherence in their reasoning or gaps in their understanding, meaning that these activities may provide cues that are more valid and diagnostic for judging understanding (Redford et al., 2012; Schleinschok et al., 2017; Thiede et al., 2010, 2022; Wiley, 2019).

Several studies have found improvements in metacomprehension accuracy when readers are prompted to generate drawings, sketches, or concept maps from unillustrated science texts. Thiede et al. (2010) tested for benefits from generating concept maps in a sample of college students enrolled in remedial reading courses. Their findings suggested that constructing concept maps while reading a set of texts on five different science topics improved both relative accuracy (the ability to differentiate between texts that were understood well from those that were understood poorly, computed as an intraindividual correlation between a participant’s predicted and actual test performance) as well as reduced overconfidence compared with students who did not engage in concept mapping. In a follow-up study with middle school students, Redford et al. (2012) also found improved relative accuracy for middle school students who generated concept maps during a second reading of a set of texts compared with students who only engaged in re-reading or who were provided with concept maps while reading. Similarly, van Loon et al. (2014) found improved relative accuracy for middle school students who were prompted to fill in concept-map templates after reading all texts, but before judging their understanding of each topic. A delayed concept-map activity helped readers to better assess their own level of understanding compared with a no-concept-map activity condition. Finally, instead of concept-mapping activities, Thiede et al. (2022) investigated differences in relative accuracy between two types of drawing activities versus a no-drawing condition. They found that when fifth grade students were prompted to create drawings during reading that reflected key causal concepts or relations from the nonillustrated texts, then this improved relative accuracy. In contrast, creating drawings that merely depicted the objects described in the non-illustrated texts did not lead to improved relative accuracy compared with the no-drawing control.

## The present study

Prior work offers concerns that the presence of illustrations in textbook excerpts may undermine metacomprehension accuracy, but also a number of promising findings suggesting that drawing activities may improve metacomprehension accuracy. The main research questions tested in this study were as follows:

- RQ1) Does the presence of illustrations for some or all textbook excerpts within a set of readings for a geoscience course improve or undermine relative metacomprehension accuracy (i.e., readers’ ability to monitor their comprehension across a set of topics)?
- RQ2) Does sketching while reading improve relative metacomprehension accuracy?

The materials were textbook excerpts and illustrations on six geoscience topics. Participants either read a set of 6 textbook excerpts that each contained a content-relevant illustration, the same set of six excerpts without the illustrations, or a set where only half of the excerpts contained the illustrations. The last condition which varies an obvious feature of the texts within the set has not yet been tested in any empirical studies of metacomprehension—even though it represents the most ecologically valid context (i.e., textbooks do not include illustrations for every topic), and is theoretically the most likely to show effects on relative accuracy. In a fully crossed design, half the participants were asked to sketch while reading.

If JOUs are driven by invalid features of illustrations, then the presence of illustrations should have a negative impact on relative accuracy. Moreover, if JOUs are based in the variable presence of illustrations more so than the variable quality of a reader’s mental model for each topic, then illustrations should have the largest negative impact on relative accuracy in the condition where only some excerpts contain illustrations. This would result in a main effect for illustration condition on relative accuracy.

Because sketching prompts the reader to generate and develop a coherent situation model about the processes explained by the text, the situation-model approach to metacomprehension predicts that it should help readers to use more valid and diagnostic cues as the basis for their JOUs, which should improve relative accuracy. This would result in a main effect for sketching condition. Sketching should also help readers to avoid misperceptions of understanding that may be prompted by the presence of illustrations, which would result in an interaction between the illustration manipulation and the sketching manipulation.

Although the main focus of the study was exploring the effects of illustrations and sketching activities on relative accuracy, the effects on both manipulations on JOUs and comprehension test scores are also reported. Exploratory analyses tested for differences in overconfidence, coded the quality of the sketches that students generated, and categorized the types of cues that students reported using to make their JOUs.

## Method

### Participants

Participants were 220 college students at a large, diverse public university who had not previously taken an introductory college course in geoscience. Students participated for course credit. Of this sample, data from 32 participants could not be analyzed due to invariant ratings (which prevents the computation of relative accuracy) and missing data. The cases that could not be analyzed were randomly distributed across the 2 × 3 design, χ^2 ^(2, *N* = 32) = 1.79, *p* = .43. The number lost in each condition were: no illustrations, no sketching 4; sketching 7; some illustrations, no sketching 2; sketching 7; all illustrations, no sketching, 6; sketching 6.

The remaining 188 cases were analyzed (62% women, 32% men, 5% preferred not to report; average age *M* = 18.89 years, *SD* = 1.18). Demographic and background information was collected in a final postactivity survey. The self-reported racial profile was 29% Asian, 11% Black/African American, 32% Hispanic, 17% White/Caucasian and 10% Other. Scores on the ACT (a standardized test of college preparedness) were available for *N* = 156. No differences were found between conditions in self-reported prior knowledge of the geoscience topics, *F*(5, 182) = 0.79, *MSE* = 1.73, *p* = .559, or ACT scores, *F*(5, 150) = 0.94, *MSE* = 20.76, *p* = .457.

Prior work has shown large effects of concept mapping activities on relative metacomprehension accuracy (Thiede et al., 2010, *d* = 1.08; Thiede et al., 2022, *d* = 1.32; van Loon et al., 2014; *d* = 0.75), but only medium size effects from prior work varying the presence of illustrations (Jaeger & Fiorella, 2024, *d* = 0.45). An a priori power analysis conducted using G*Power Version 3.1.9.2 and the more conservative medium effect size (*f* = 0.25), indicated the minimum sample size needed to achieve 80% power in detecting main effects and interactions at a significance criterion of α = 0.05 in a between-subjects ANOVA on relative accuracy was approximately 30 per condition (*N* = 158). We oversampled with the goal of reaching at least 80% power after losing participants due to invariant judgments and missing data, which is not known until analysis.

### Materials

The materials (https://osf.io/qkyv7/) were excerpts and illustrations from the textbook used for the introductory college geoscience course at our university (Marshak, 2012) on six topics (weathering of rocks, uplift and mountain formation, coal formation, wave movement, cave formation, and El Niño). The textbook excerpts were written at the 11th grade level (Flesh-Kincaid), and were approximately 950 words each. The illustrations were full-color, multipaneled composites that contained a mixture of photographic, realistic depictions and more abstract diagrammatic content, with labels for parts of objects and processes.

To test understanding from the readings, five multiple-choice questions on each topic (available on the OSF site) were developed in collaboration with a geoscience expert and educator who teaches introductory geoscience courses. Answering these questions required the reader to make inferences and reason about the processes that were described, and did not rely simply on verbatim memory for the information that appeared in the texts. For example, below is an inference question that relies on understanding the El Niño excerpt. Anchovies are not mentioned in the text, and answering this question requires understanding the effects of El Niño on upwelling, water temperature, air pressure, and rain, which are all mentioned separately in the text:

Anchovies are cold water fish that live near the ocean surface off of Peru and Ecuador in the equatorial eastern Pacific. During which weather patterns will anchovies do best?

a. when there is more rain in the equatorial western Pacific*

b. when there is more upwelling in the equatorial western Pacific

c. when there is higher air pressure in the equatorial western Pacific

d. when trade winds weaken

As part of materials development, a small sample (*N* = 5) completed the test questions with the text available. For each question, the majority was able to select the correct answers and overall performance was 92% correct. This shows that the low performance in the main study is not because the questions could not be answered from the readings.

The presence of illustrations was manipulated so that all, none, or some (half, counterbalanced) of the excerpts had illustrations. In the all-illustrations condition, textbook images were presented with all of the excerpts. In the no-illustrations condition, none of the images were presented. Two versions of the some-illustrations condition were created in which half of the topics were illustrated, counterbalanced across the two sets.

### Procedure

All procedures took place in an in-person, experimenter proctored session. The basic procedure followed the standard design in metacomprehension studies of having participants read, judge, and take tests on a set of texts (Glenberg & Epstein, 1985; Maki & Berry, 1984). In this study, participants were asked to study the texts as they would normally do for a class. After reading each text, participants provided JOUs by predicting how many questions they would get correct on a quiz with five questions for each topic (0–5 scale). Before starting the main task, they were informed about the kind of test questions they should expect, based on past work showing that clarifying the nature of the upcoming test questions is important for helping readers to judge their understanding of a topic (Griffin et al., 2019b). Participants were informed “The literature suggests that people study differently depending on the kind of tests they expect. You will be taking tests that assess your ability to make connections between the different parts of a text (i.e., link the parts of the text).” Then they were shown an example text on lightning formation, and answered two practice inference questions. After answering these questions, they were told “The practice tests you just took had items that required that you go beyond what the text said and identify correct inferences implied by the text. You were tested on how well you could make connections among different parts of the text you read. The questions asked you about how or why processes like lightning happen. As you read this set of textbook excerpts, your goal is to understand how and why processes are happening so that you can answer these types of test questions.”

After reading the example text and answering the practice questions, participants proceeded to the main study. First, they read the six geoscience texts and made JOUs after reading each text. Then, participants completed the comprehension tests with five questions for each topic. All topics appeared in the same order for all participants and across each of the tasks. All tasks were untimed.

The excerpts were presented as pdf pages on a desktop computer. Half of the participants read without sketching. The other half were instructed that as they read they should draw a sketch that conveyed the important concepts about the processes explained by the excerpt (i.e., draw a sketch about the different processes that break up rock into sediment). They were provided with paper booklets with separate blank pages to create their sketch for each excerpt. They were told that the sketches did not need to be artistic or realistic, and could just be boxes and arrows. When they finished reading, they advanced to a new screen on the computer so that the text was no longer available, and were asked to turn the page in the paper booklet before making their JOUs on the computer. They did not have access to the texts or their sketches during the testing phases. After taking all six tests, they were asked to describe the basis that they used to make their judgments with this question prompt: “When you predicted your performance on the tests for each topic after reading, what did you consider?”.

Following the study, participants completed a final survey in which they were asked for demographic information and to self-report their knowledge of the topics on a 1–7 point scale (How much do you feel you knew about each of these topics before doing this study?) and ACT scores. The final survey also assessed whether they possessed a multimedia superiority belief by asking them to indicate whether they believed they learn better with text and images or text alone. Consistent with prior work on the multimedia belief heuristic (Serra & Dunlosky, 2010), most students endorsed the belief that they learn better from multimedia. Only eight participants indicated that they believed they learned better with text alone. The results do not change if they are excluded from analyses.

### Dependent measures

The main dependent measure of interest was relative accuracy. Relative accuracy was computed as the intraindividual correlation between each participant’s JOUs and their comprehension test performances across the set of topics. For this intraindividual correlation measure, higher values reflect more accurate comprehension monitoring (better discrimination of what is less vs. more understood). Pearson correlations were used because both predictions and scores ranged from 0 to 5 (rather than 0,1 data for which gamma correlations are more appropriate, see Griffin et al., 2019a, for a detailed explanation).^1^ Average JOUs and average scores on the comprehension tests are also reported as proportion scores.

Exploratory analyses tested for differences in confidence bias. Confidence bias was computed as the difference between JOUs and actual test performance (positive values mean overconfidence, and more extreme values represent greater illusions of understanding). Other exploratory analyses looked at the cue-basis responses. Following coding done by Jaeger and Wiley (2014), and Wiley et al. (2018), responses were coded into comprehension-based and non-comprehension-based categories. One category reflected the use of heuristics based in superficial text features (topic familiarity, interest, length, vocabulary) or memory-based cues. If the student referred to their ability to remember the text, or their interest, prior knowledge, or familiarity with the topics, then the student was also coded as having used non-comprehension-based cues. If students mentioned their ability to explain, teach, connect ideas, get the point, comprehend or understand the topics, then the student was coded as having used comprehension-based cues. In this study, when students mentioned making use of the images, their drawings, and their notes to assess how much they understood each text, these were also coded as comprehension cues (e.g., pictures helped me understand the topic better, how well I sketched out the notes, how much thought I put into the notes, how much I sketched for each topic). Cohen’s Kappa of two independent coders was .82.

**Fig. 1:**
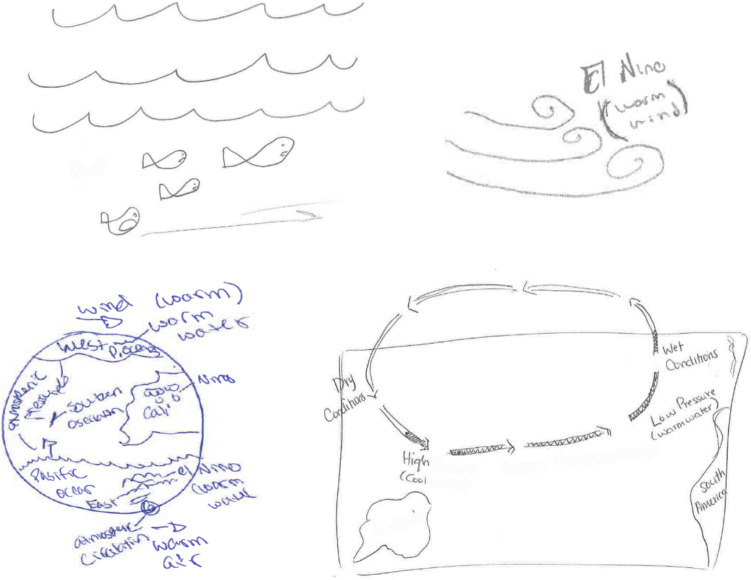
Example student sketches from El Nino excerpt

Exploratory analyses were also done on sketch quality. Following the manipulation used in Thiede et al. (2022), the sketches were coded for whether they were depictive (showing objects) versus explanatory (including key causal concepts, relations, or comparisons described in the text). Cohen’s Kappa of two independent coders was .93. Examples of the kinds of sketches that students generated are included in Fig. 1. The top two are examples of depictive images, the bottom two represented causal concepts and relations.

### Data availability

The experiment was not preregistered. The data and analyses outputs are available online (https://osf.io/qkyv7/).

## Results

### Relative accuracy

**Fig. 2:**
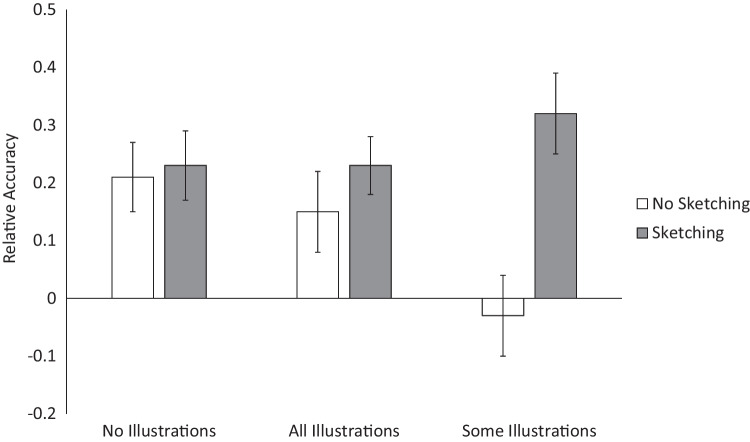
Effects of sketching and illustration conditions on relative accuracy. Error bars represent ± 1 *SEM*

As shown in Fig. 2, although relative accuracy was low overall (mean *r* = 0.19), there were differences in relative accuracy across conditions. An ANOVA on relative accuracy revealed no main effect for illustration condition, *F*(2, 182) = 0.72, *MSE* = 0.13, *p* = .486, but there was a significant benefit from sketching, *F*(1, 182) = 9.16, *MSE* = 0.13, *p* = .003, η_p_^2^ = .048. In addition, there was a significant interaction, *F*(2, 182) = 3.30, *MSE* = 0.13, *p* = .039, η_p_^2^ = .035.

To follow up this interaction, relative accuracy was examined for each sketching condition separately. With sketching, relative accuracy was similar across the three illustration conditions, *F*(2, 91) = 0.54, *MSE* = 0.12, *p* = .587. In contrast, in the no-sketching condition there was a significant effect of illustration condition, *F*(2, 91) = 3.16, *MSE* = 0.15, *p* = .047, η_p_^2^ = .065. Pairwise Tukey’s HSD comparisons indicated that monitoring accuracy was significantly worse when only some topics were illustrated than with no illustrations, *p* = .048, *d* = 0.62. Relative accuracy when all topics were illustrated fell in between the other conditions, but was not significantly different from either. The same main effect for sketching and interaction were found when ACT scores were used as a covariate.

### JOUs and test scores

The average JOUs and test scores (and *SD*s) in each condition are reported in Table 1. The subscores for the illustrated and non-illustrated topics are broken out and reported for the condition that contained both kinds of texts (the some-illustrations condition).

**Table 1:** Average means and SDs for JOUs, test scores and overconfidence, and frequency of comprehension-based cue use by sketching and illustration conditions

	JOUs	Test Scores	Overconf	Comp Cues	*N*
No Sketching					
No Illustrations	.54 (.16)	.41 (.12)	.14 (.20)	.60	30
All Illustrations	.50 (.16)	.34 (.11)	.16 (.19)	.33	33
Some Illustrations	.44 (.13)	.33 (.11)	.11 (.17)	.35	31
Not Illustrated	.45 (.15)	.31 (.09)	.14 (.18)		
Illustrated	.42 (.11)	.34 (.12)	.08 (.16)		
Sketching					
No Illustrations	.49 (.14)	.41 (.13)	.08 (.18)	.52	31
All Illustrations	.52 (.12)	.38 (.13)	.14 (.18)	.77	30
Some Illustrations	.56 (.20)	.42 (.14)	.14 (.19)	.72	33
Not Illustrated	.56 (.22)	.39 (.14)	.16 (.19)		
Illustrated	.57 (.20)	.45 (.14)	.12 (.19)		

*JOU*, Judgment of Understanding; *Overconf,* Overconfidence; *Comp Cues*, Proportion of Participants Who Reported Using Comprehension-based Cues; *N*, Number of Participants. Within the ‘Some Illustrations’ conditions, results are also reported separately for the Not Illustrated versus the Illustrated versions of the texts

An ANOVA on test scores revealed there was a significant effect of sketching on comprehension, *F*(1, 182) = 6.15, *MSE* = 0.02, *p* = .014, η_p_^2^ = .033. Scores were higher in the sketching condition. The main effect of illustration condition, *F*(2, 182) = 2.66, *MSE* = 0.02, *p* = .073, and interaction, *F*(2, 182) = 2.04, *MSE* = 0.02, *p* = .133, did not reach significance.

**Fig. 3:**
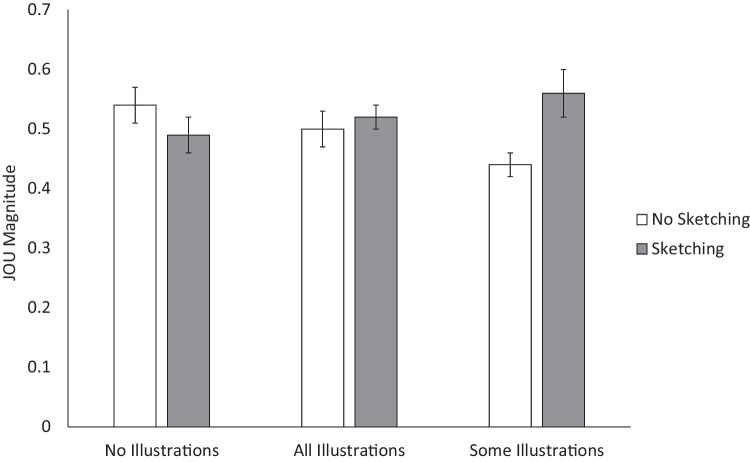
Effects of sketching and illustration conditions on JOU magnitude. Error bars represent ± 1 *SEM*. * Note*. JOU = Judgment of Understanding

As shown in Fig. 3, an ANOVA on JOUs revealed no main effect of illustration condition, *F*(2, 182) = 0.16, *MSE* = 0.02, *p* = .855. The effect of sketching was also not significant, *F*(1, 182) = 2.09, *MSE* = 0.02, *p* = .150, although there was an interaction, *F*(2, 182) = 5.11, *MSE* = 0.02, *p* = .007, η_p_^2^ = .053. Pairwise Tukey’s HSD comparisons indicated that JOUs were significantly higher in the sketching than non-sketching conditions only when some topics were illustrated, *p* = .016, *d* = 0.82.

Since no differences were seen in JOUs between the all-illustrations and no-illustrations conditions, this suggests the textbook illustrations did not simply increase or decrease perceptions of understanding. However, other similar effects (font size, Chang & Brainerd, 2023) also fail to appear when JOUs are compared between subjects. To look at whether differences might emerge when the presence of illustrations is manipulated within subjects, the JOUs for the partial illustration condition are shown in Table 1 with separate averages for the subset that included illustrations and the subset that did not. There were no differences in JOUs when images were manipulated within subjects, *F*(1, 62) = 0.14, *MSE* = 0.004, *p* = .708, nor in overconfidence, *F*(1, 62) = 1.42, *MSE* = 0.02, *p* = .238. This suggests that participants were not using a simple heuristic that uniformly inflated their JOUs for illustrated texts.

### Exploratory analyses

#### Confidence bias

Overconfidence in each condition is shown in the Overconf column in Table 1. Because the pattern of JOUs and test performance were largely similar, an ANOVA on overconfidence showed no differences either due to sketching, *F*(1, 182) = 0.24, *MSE* = 0.03, *p* = .627, or illustration conditions, *F*(2, 182) = 0.78, *MSE* = 0.03, *p* = .461, nor their interaction, *F*(2, 182) = 0.90, *MSE* = 0.03, *p* = .410. However, a one-sample *t* test versus 0 showed there was significant overconfidence across all conditions, *t*(187) = 9.47, *p* < .001, *d* = 0.69.

#### Sketch quality

All students in the sketching conditions drew or wrote something on the blank pages in their booklets. Sketch quality was coded for participants in the sketching condition (*n* = 94), with each participant receiving a separate quality code for each of the 6 topics, resulting in a dataset with 564 observations. Only 30% (171/564) of the sketches were coded as including important causal concepts, relations, or comparisons. A mixed effects model with random intercepts for participants and texts provided a good fit for the data, *F* (1, 527.66) = 13.27, *p* < .001, *R*^2^ conditional = 0.23, and showed that a fixed effect of sketch quality significantly predicted performance on the comprehension tests, *B* = 0.45, *SE* = 0.12, *t* = 3.64, *p* < .001.

#### Cue basis

The results of the cue-basis coding found that 45.2% of the participants (*n* = 85) reported using non-comprehension-based cues. The remaining participants reported using comprehension-based cues (*n* = 103), including 18.1% of the total (*n* = 34) who specifically mentioned using the images, drawings or their notes as a basis for making their JOUs. The remaining 36.7% did not mention these features. An ANOVA explored the effects of sketching and illustration conditions on the use of comprehension-based cues as the dependent variable. As shown in the Comp Cue percentage column in Table 1, sketching led to a significantly higher frequency of reporting comprehension-based cues (67%) over the no-sketching conditions (43%), χ2 (1, *N* = 188) = 11.36, *p* < .001. Similar to the interaction seen for relative accuracy, sketching increased reports of comprehension-based cues, in the all-illustrations, χ2 (1, *N* = 64) = 12.54, *p* < .001, and some-illustrations conditions, χ2 (1, *N* = 63) = 8.39, *p* = .004, but not in the no-illustrations conditions, χ2 (1, *N* = 61) = 0.43, *p* = .510. Importantly, using comprehension-based cue use as the independent variable to test for differences between groups in relative accuracy showed that readers who reported using comprehension-based cues had higher relative accuracy (*M* = .25, *SD* = .32) than those who reported using non-comprehension-based cues (*M* = .11, *SD* = .42), *t*(186) = 2.60, *p* = .010, *d* = 0.38, which suggests that sketching prompted the use of more diagnostic cues. Relative accuracy was descriptively higher for students who reported using images, drawings or their notes, *M* = .29, *SD* = .32, but this did not differ significantly from the students who reported other types of comprehension-based cues, *M* = .23, *SD* = .32,* t*(101) = 0.86, *p* = .393.

## Conclusions

The results of this study demonstrate that the presence of illustrations may undermine comprehension monitoring under some conditions. These findings were observed in the context of a large public university where there is a lot of variability in student reading skill levels and many students may struggle to understand texts written at the 11th grade level. It is possible that the difficulty of the texts and/or the lack of advanced comprehension skills may have contributed to poor monitoring. Yet, within this sample, the context that led to the poorest relative accuracy was when only some topics were presented with illustrations and some were not. This result highlights an important source of difficulty for engaging in accurate monitoring in real course contexts, as it is unlikely that textbooks will include illustrations for every topic. The fact that the all-illustration condition fell in between the other conditions is consistent with prior research that has found only weak and inconsistent effects on relative accuracy when all texts include illustrations (Jaeger & Fiorella, 2024; Jaeger & Wiley, 2014).

A second important result was that sketching while studying improved relative accuracy overall, and appeared to eliminate the misperceptions of comprehension caused by the varying presence of images. This finding is consistent with the predictions of the situation-model approach and extends prior work showing benefits of generative activities as a way of providing readers with a valid basis for making accurate judgments of metacomprehension, including activities that involve explanation (Fukaya, 2013; Griffin et al., 2008, 2019b; Jaeger & Wiley, 2014; Wiley et al., 2016a), concept mapping (Redford et al., 2012; Thiede et al., 2010; van Loon et al., 2014) and drawing (Schleinschok et al., 2017; Thiede et al., 2022; Wiley, 2019). Importantly, the sketching instruction used here was similar in spirit to the drawing instruction used by Thiede et al. (2022) which focused readers on creating a representation of the process that was being explained and not merely a depiction of the objects that were described. Although the current results are consistent with prior ones, it may also be important to note that students generally received more support in previous studies on concept mapping activities. It is possible that even more robust effects could be seen if students have the opportunity to practice sketching, are instructed how to generate drawings (Redford et al., 2012; Thiede et al., 2010), or are provided with templates (van Loon et al., 2014). This may be one reason why relative accuracy was generally higher in prior concept mapping studies.

Even though most of the participants endorsed holding the multimedia superiority belief, the lack of an illustration effect on JOU magnitude showed that JOUs did not reflect a general application of that belief as a heuristic cue. This is consistent with prior work using a similar methodology (Jaeger & Fiorella, 2024; Jaeger & Wiley, 2014; Wiley et al., 2017), but differs from the general illusion of understanding due to images that has been found in studies where a single topic was used and an image accompanied every few sentences of text (Ikeda et al., 2013; Serra & Dunlosky, 2010), or when images are not accompanied by text (Caldwell et al., 2017). The current results suggest that images impair relative accuracy in a more idiosyncratic, reader-specific way. For each reader, each image might help, harm, or have no effect on their comprehension. Likewise, each reader may view a particular image as helping, harming, or having no effect on their comprehension. What matters for relative accuracy is that for a particular person, the impact on their comprehension from text to text and image to image is similar to the impact on their judgments of comprehension. Sketching appears to improve that alignment for individual readers.

Other findings were generally consistent with prior work. First, sketching supported better understanding of the texts. This finding is consistent with the notion that sketching may promote more constructive processing and active integration of information. Second, the presence of the illustrations did not generally lead to better understanding of the texts (Butcher, 2006; Jaeger & Fiorella, 2024; Renkl & Scheiter, 2017). One possible reason for this could be the complexity of the textbook illustrations, which generally consisted of composite images that combined realistic and diagrammatic elements. Prior work by Butcher (2006) has noted that complex images can be problematic for learners. In this previous work, complexity of images was closely related to their realism—the degree to which they faithfully represent the actual process or entity in question. This was contrasted with more simplified or abstracted diagrams. For example, to depict the mechanism of blood flow within the heart, a complex photorealistic heart could be displayed (which necessarily contains many features), or a simplified diagram could be created which strips the image of its visual complexity and reduces it to the bare visual information needed to convey the abstract process of blood flow. In the Butcher study, the simplified diagrams more strongly supported information integration and deeper comprehension processes during learning.

Another possible reason why the textbook illustrations did not support better understanding could be because some students may have been processing the images only superficially, and failed to use the images to guide their understanding of the text. Or perhaps some portions of the illustrations were helpful but other aspects were distracting. A related explanation for poor monitoring would be that readers were picking up on idiosyncratic features of the images, or were attending primarily to realistic or photographic elements rather than processes depicted by abstract or symbolic diagrams (Wiley et al., 2017). Especially when readers were not engaging in sketching, these tendencies could lead readers to use invalid cues as the basis of their JOUs. Unpacking the role of the complexity and realism in the textbook images in undermining relative accuracy outcomes will be an interesting issue to pursue in future studies.

Another result that is generally consistent with prior work was the low level of relative accuracy seen in this course context. This matches a growing number of studies that have demonstrated lower relative metacomprehension accuracy in authentic learning contexts using materials from a single course or textbook, where there is greater similarity or conceptual overlap among the topics (Hildenbrand et al., 2024). Most research on metacomprehension has been conducted using sets of texts on different topics, and reviews of metacomprehension results have found average intraindividual correlations between predictions and performance of around 0.27 for adult readers (Dunlosky & Lipko, 2007; Griffin et al., 2019a; Maki, 1998b; Thiede et al., 2009; Yang et al., 2023). In contrast, studies conducted using sets of texts from the same textbook, or sections of a longer passage, have observed lower levels of relative accuracy, with intraindividual correlations between predictions and performance on comprehension tests approaching zero (Griffin et al., 2009; Guerrero et al., 2022, 2024; Maki et al., 1994; Maki, 1998a; Ozuru et al., 2012; Wiley et al., 2016a). The instruction to draw a sketch of the processes may have helped to make these topics more distinct in readers’ minds and appears to have helped readers to have a better sense of their understanding of each one. The results are consistent with a recent study using a set of biology texts. Fiorella and Jaeger (2023) found similar results of low overall relative accuracy and modest increases when students engaged in generating drawings while reading. In another recent study using a set of psychology texts, Griffin et al. (2025) found low overall relative accuracy, and that explanation activities led to small and sometimes non-significant increases. The lack of substantial improvement in relative accuracy from interventions that have been effective with more diverse sets of topics might indicate that an instruction that is sufficient in laboratory contexts may be too minimal for real course application.

Nevertheless, these results have educational implications for science learning. Sketching-based activities may offer a simple but effective means of promoting both more accurate comprehension monitoring as well as improved scores on assessments of comprehension. Although relative accuracy was fairly low overall (as noted above), this likely stems from the similarity and complexity of the geoscience topics. Despite the difficulties for comprehension and monitoring created by this, sketching still produced higher scores relative to the other conditions. Sketching activities may be particularly useful in courses such as geoscience where visualizations are thought to be critical for understanding, but it is also possible that sketching may be helpful whenever science textbooks and other learning materials contain a mix of both text-only content and multimedia content. The next steps for this research include exploring questions like these, as well as the conditions that may allow for further improvements in monitoring accuracy when students are faced with the difficult task of discriminating their understanding of texts on overlapping topics.

## Data Availability

The data and materials are available online (https://osf.io/qkyv7/). The experiments were not preregistered.

## Footnotes

1 Relative accuracies computed using gamma show similar patterns (available in data set on OSF).

## Code availability

N/A.
